# Comparative efficacy of lithium carbonate loading dose versus standard dosing, each combined with quetiapine, in acute mania of bipolar I disorder: a randomized single-blind controlled trial

**DOI:** 10.1186/s12888-026-08084-z

**Published:** 2026-04-21

**Authors:** Ebrahim Moghimi-Sarani, Seyedeh Zahra Etemad, Raziye Dehbozorgi

**Affiliations:** 1 Department of Psychiatry Research Center for Psychiatry And Behavioral Science, Shiraz University of Medical Sciences, Shiraz, Iran; 2 Community Based Psychiatric Care Research Center, School of Nursing and Midwifery, Shiraz University of Medical Sciences, Zand St, Shiraz, 713481436 Iran

**Keywords:** Lithium carbonate, Quetiapine fumarate, Bipolar disorder, Mania

## Abstract

**Introduction:**

Bipolar disorder type I is defined by the occurrence of at least one manic episode, presenting with elevated mood, hyperactivity, and restlessness. Effective management relies primarily on pharmacotherapy, with lithium carbonate and quetiapine proven beneficial as monotherapies. Given the chronic nature of the illness and alternating manic–depressive phases, acute mania requires rapid symptom control to prevent functional decline and harm. Although lithium is a cornerstone mood stabilizer, its clinical effect may take days to emerge. Quetiapine, an atypical antipsychotic, provides faster relief in acute mania. Evidence suggests that combining these agents may offer superior control of manic symptoms compared to either drug alone.

**Objective:**

This study aims to compare the efficacy of a lithium carbonate loading dose combined with quetiapine against the standard lithium carbonate dosage alongside quetiapine for managing acute mania.

**Methods:**

This interventional randomized clinical trial (IRCT20181204041847N2) was a prospectively designed, retrospectively registered study conducted at a psychiatric hospital and conducted on 60 adult inpatients diagnosed with Bipolar I disorder in the acute manic phase, according to DSM-5 criteria and a Young Mania Rating Scale (YMRS) score of ≥ 20. Participants were equally allocated to two arms using block randomization. The loading-dose lithium group received a loading dose of lithium carbonate on Day 1 at 20 mg/kg (maximum 1,800 mg), followed by standard dosing from Day 2 onward. The standard-dose lithium group received lithium carbonate starting at 300 mg/day, increased in 300 mg increments every 2–3 days until reaching 900–1,200 mg/day, with serum concentrations consistently maintained within 0.6–1.2 mEq/L. Quetiapine was initiated at 100 mg/day in two divided doses and titrated by 100–200 mg/day increments to a target of 400–800 mg/day based on clinical response and tolerability. Mania severity was assessed using the YMRS at baseline and on Days 3, 7, and 14 after treatment initiation.

**Results:**

In this study of 60 patients with acute mania, both treatment groups showed significant reductions in YMRS scores over 14 days (*p* < 0.001). The loading dose group achieved a greater mean decrease from baseline (− 17.70 ± 3.76) compared with the standard dose group (− 13.96 ± 2.94; *p* < 0.001). Lithium serum levels reached therapeutic ranges sooner in the loading group (Day 5: 0.82 ± 0.11 mEq/L vs. 0.65 ± 0.13; Day 10: 0.78 ± 0.09 vs. 0.68 ± 0.10). Aggressive/disruptive behavior scores were also lower in the loading group by Day 14 (0.36 ± 0.06 vs. 0.89 ± 0.53; *p* = 0.012). The incidence of common adverse events was comparable, and no cases exceeded lithium serum levels of 1.4 mEq/L.

**Conclusion:**

The study found that adding a lithium loading dose to quetiapine significantly enhanced the reduction of acute mania symptoms compared with standard dosing, without increasing short-term adverse events. These results support the regimen’s clinical benefit, though confirmation through larger, long-term trials is needed.

**Clinical trial number:**

IRCT20181204041847N2; Registration Date: 16 August 2023; No: 68,388.

## Introduction

Bipolar disorder type I is a prevalent and disabling psychiatric condition with a lifetime prevalence close to 1%, often presenting between the ages of 20 and 25. Early onset correlates with a less favorable long‑term outcome due to recurrent manic and depressive episodes affecting social and occupational functioning. Globally, bipolar disorder ranks among the most disabling illnesses and poses substantial socioeconomic burdens. The acute manic phase requires rapid intervention to mitigate risks of impaired judgment, impulsive actions, and hospitalization [1, 2].

The acute manic phase is characterized by persistently elevated mood, increased energy, inflated self-esteem, rapid or pressured speech, and reduced need for sleep. These symptoms often result in marked deficits in functioning, compromised judgment, and a heightened propensity for impulsive or hazardous actions. Timely and effective management of mania is essential to ensure patient safety, prevent symptom escalation, shorten hospitalization, and promote participation in longer-term strategies for mood stabilization [3].

For decades, lithium carbonate has served as the cornerstone pharmacological therapy for bipolar disorder, with robust evidence supporting its efficacy in the treatment of acute mania, prophylaxis against manic and depressive relapse, and reduction of suicide risk. Its multifaceted mechanism of action encompasses modulation of diverse neurotransmitter systems, regulation of intracellular signaling cascades, and potential neuroprotective effects [4].

However, lithium has a relatively slow onset of action, with therapeutic effects often taking several days to become apparent. This delay can be a significant limitation in managing severe or rapidly escalating manic episodes where immediate symptom relief is paramount. To accelerate therapeutic lithium levels, loading dose strategies have been explored, aiming to reach therapeutic serum concentrations more quickly [5].

Quetiapine, a second-generation antipsychotic, has established itself as an important therapeutic agent for acute manic episodes, effective both as monotherapy and in combination with mood stabilizers. Its pharmacological activity is mediated through antagonism of multiple neurotransmitter receptors, including dopamine D2 and serotonin 5-HT2A receptors—pathways that play a central role in the neurobiology of mania [5–7].

Quetiapine has shown rapid and clinically significant efficacy in alleviating manic symptoms, with a safety profile that is generally well-tolerated. Notably, it is associated with a lower incidence of extrapyramidal adverse effects compared to many first-generation antipsychotics, contributing to its favorable acceptance in the acute management of mania [3].

The potential for synergistic effects between lithium and quetiapine has been recognized, given their distinct but complementary mechanisms of action [3]. Lithium’s long-term mood-stabilizing properties and potential neuroprotective effects, combined with quetiapine’s robust acute antimanic and anxiolytic properties, suggest a promising therapeutic combination. While standard dosing of both agents is commonly employed, the optimal strategy for initiating treatment, particularly concerning lithium dosing, remains an area of investigation. The combination of lithium and quetiapine offers potentially synergistic benefits owing to complementary mechanisms of action. Evidence from randomized trials indicates quetiapine may achieve faster symptomatic improvement than lithium alone in early‑course patients, supporting exploration of dosing strategies that optimize both agents’ strengths [4, 6].

This study aims to address this by comparing the effectiveness and safety of a rapid lithium loading dose strategy in combination with standard quetiapine dosing against a standard lithium titration strategy with standard quetiapine dosing in patients with acute mania [3, 5, 8].

The rationale for comparing loading versus standard lithium protocols in combination therapy stems from the distinct pharmacokinetic and pharmacodynamic profiles of lithium and quetiapine. Quetiapine is known for its rapid central effects, quickly modulating neurotransmitter systems involved in mania. In contrast, lithium’s mood-stabilizing and neuroprotective effects are often associated with slower onset, requiring time for intracellular adaptations [9, 10]. By combining quetiapine’s rapid action with a strategy to accelerate lithium serum levels, the study aimed to investigate whether this could lead to faster and more robust symptom control in the acute phase of mania.

## Methods

### Study design and setting

This study was prospectively designed but retrospectively registered and was conducted between 2022 and 2023 at the Department of Psychiatry, Ibn Sina Hospital in Shiraz, a tertiary psychiatric care center. A post-hoc power analysis (G*Power 3.1) with α = 0.05, an effect size of 0.8, and a sample size of 60 indicated 98% power to detect intergroup differences in YMRS scores of ≥ 4 points (25).

The study protocol received ethical approval from the Institutional Review Board of Shiraz University of Medical Sciences (Approval Number: IR.SUMS.MED.REC.1401.530) and was registered in the Iranian Registry of Clinical Trials (IRCT20181204041847N2; Registration Date: 16 August 2023; No: 68388). Written informed consent was obtained from all participants following a thorough explanation of the study aims, procedures, potential risks, and anticipated benefits.

This was a randomized single-blind controlled interventional trial in which both arms received active treatment regimens. Participants, treating psychiatrists, and outcome assessors were blinded to treatment allocation, while nursing staff administering medications were necessarily unblinded to ensure accurate dosing, particularly in distinguishing between the lithium-loading and standard dosing regimens. Both patients and their caregivers received identical medication schedules to maintain blinding integrity.

### Participants

Adult patients (aged 18–65 years) consisted of male and female participants (37 males and 23 females) diagnosed with bipolar I disorder, current manic episode, according to the Diagnostic and Statistical Manual of Mental Disorders, Fifth Edition (DSM-5) criteria, were eligible for inclusion. Patients were required to have a Young Mania Rating Scale (YMRS) score of ≥ 20 at screening and baseline to confirm the presence of moderate to severe mania.

#### Exclusion criteria included

Diagnosis of schizoaffective disorder, schizophrenia, or other psychotic disorders. Current or recent (within 4 weeks) use of electroconvulsive therapy (ECT). History of lithium intolerance or contraindications. Current use of other psychotropic medications that could interfere with study assessments, except for stable doses of benzodiazepines for agitation (which were allowed if initiated prior to study entry and maintained at a constant dose). Significant hepatic or renal impairment (serum creatinine > 1.5 mg/dL or AST/ALT > 3 times the upper limit of normal). Pregnancy or breastfeeding. Any medical condition that, in the opinion of the investigators, would preclude safe participation. Serum lithium level > 1.4 mEq/L at baseline prior to study initiation.

### Study interventions

Both study arms were designed as interventional protocols with active pharmacological treatments. Participants were randomly assigned in a 1:1 ratio to one of two treatment groups using a computer-generated randomization sequence:

#### Loading-dose lithium group

##### Lithium carbonate

A loading dose of 20 mg/kg of elemental lithium was administered orally on Day 1, with a maximum total dose of 1,800 mg. This was followed by a standard lithium carbonate titration, aiming for a serum level between 0.6 and 1.2 mEq/L.

##### Quetiapine

Initiated at 100 mg/day, with subsequent titration by 100–200 mg/day increments, based on clinical assessment and tolerability, to a target therapeutic dose range of 400–800 mg/day. The daily dose was divided into two administrations.

#### Standard-dose lithium group

##### Lithium carbonate

Standard-dose titration of lithium carbonate was initiated at 300 mg/day and increased by 300 mg every 2–3 days, aiming for a serum level of 0.6–1.2 mEq/L. The Standard dose was 900–1,200 mg/day, adjusted to serum levels 0.6–1.2 mEq/L at days 5 and 10.

##### Quetiapine

Initiated at 100 mg/day in two divided doses, with gradual titration by 100–200 mg/day every 1–2 days to a target range of 400–800 mg/day. The specific dose was determined according to patient symptom severity, observed clinical improvement, and tolerability (Table 2).

### Study procedures

All participants were admitted to the inpatient psychiatric unit for the duration of the 14-day study period.

### Medication management and monitoring

#### Lithium monitoring

Serum lithium levels were measured at baseline, Day 2, Day 5, Day 10, and Day 14. Doses were adjusted by the treating physician to maintain serum lithium levels within the therapeutic range of 0.6–1.2 mEq/ L. Doses were adjusted based on the most recent serum lithium level and clinical assessment.

#### Quetiapine dosing

Quetiapine was initiated at 100 mg/day in two equally divided doses. Dose escalation proceeded by 100–200 mg/day increments every 24–48 h, aiming for a final therapeutic range of 400–800 mg/day. Dose adjustments within this range were individualized according to baseline YMRS score, rate of symptom reduction, and side-effect profile.

#### Vital signs and clinical assessment

Vital signs (blood pressure, heart rate, respiratory rate, temperature) were monitored every 12 h for vital signs and daily for symptom review. Mental status examinations were performed daily by the treating psychiatrist.

### Safety monitoring

Adverse events were assessed and recorded daily symptom checklist plus patient spontaneous report. Patients were asked about common side effects including nausea, vomiting, diarrhea, tremor, thirst, polyuria, sedation, dizziness, dry mouth, weight changes, and any other discomfort. Any serious adverse events (SAEs) were recorded and reported to the IRB and regulatory authorities as required. ECGs were performed at baseline and Day 7 to monitor for potential QTc prolongation, although quetiapine’s risk is generally low.

### Exclusion for high lithium levels

The exclusion for lithium ≥ 1.4 mEq/L applies post-dosing as a safety measure. If any patient’s serum lithium level exceeded 1.4 mEq/L at any point during the study after initial dosing, they were closely monitored. If the level persisted or caused significant toxicity, the lithium dose would be reduced or discontinued, and they would continue on quetiapine alone or with an alternative mood stabilizer at the discretion of the treating physician, but their data would be analyzed according to their assigned group up to the point of discontinuation or modification if within the protocol’s definition of per-protocol analysis.

### Outcome measures

#### Primary outcome

Change in YMRS score from baseline (Day 0) to Day 14.

#### Secondary outcomes

Change in Clinical Global Impression-Severity (CGI-S) score from baseline to Day 14. Change in Clinical Global Impression-Improvement (CGI-I) score at Day 14. Incidence and severity of adverse events. Proportion of patients achieving a ≥ 50% reduction in YMRS score from baseline at Day 14. Proportion of patients achieving a YMRS score ≤ 12 at Day 14 (indicating minimal symptoms).

### Statistical analysis

Data were analyzed using IBM SPSS Statistics for Windows, Version 24.0 (IBM Corp., Armonk, NY, USA). Descriptive statistics (mean, standard deviation, frequencies, percentages) were used to summarize demographic and baseline characteristics, as well as outcome measures. Due to the complete dataset and the nature of the outcome measures (continuous variables assessed over time), A repeated-measures ANOVA was preferred over a mixed-effects model for the primary and secondary efficacy endpoints. This approach allows for the examination of within-subject changes over time and between-group differences at specific time points, while accounting for the correlated nature of repeated measurements. An intention-to-treat (ITT) analysis was performed, including all randomized participants. For missing data, a last-observation-carried-forward (LOCF) approach was specified for the primary analysis to preserve the intention-to-treat principle. However, no participants withdrew, and no assessment points were missing during the 14-day study; therefore, the LOCF strategy was not ultimately required. Significance level was set at α = 0.05.

## Results

### Participant flow and baseline characteristics

A total of 60 patients were enrolled in the study. 30 patients were randomized to the loading-dose lithium group, and 30 to the standard-dose lithium group. All 60 patients completed the 14-day study period, with no dropouts and no exclusions due to serum lithium levels > 1.4 mEq/L.

Baseline demographic and clinical characteristics in Table 1 were generally comparable between the loading-dose and standard-dose lithium groups, with no significant differences in most variables, including number of children, prior hospitalizations, sex distribution, marital status, education, and place of residence. A significant difference was observed in occupational status (*p* = 0.0178), with more unemployed participants in the loading-dose lithium group, and baseline YMRS scores were slightly higher in this group (32.06 ± 6.97 vs. 28.96 ± 5.30; *p* = 0.057). These imbalances should be considered as confounders in interpreting the outcomes.

**Table 1:** Baseline demographic and clinical characteristics of the loading-dose and standard-dose lithium groups

Variable	Categories	Standard-dose lithium group (*n* = 30)	Loading-dose lithium group (*n* = 30)	*p*-value
Number of children	Mean ± SD	1.33 ± 1.30	1.73 ± 1.02	0.262
Hospitalizations	Mean ± SD	3.00 ± 1.85	3.36 ± 2.22	0.588
Sex	Male	18 (60%)	19 (63.3%)	> 0.999
	Female	12 (40%)	11 (36.7%)	
Marital Status	Married	11 (36.7%)	13 (43.3%)	0.097
	Single	18 (60%)	12 (40%)	
	Widowed/Divorced	1 (3.3%)	6 (20%)	
Education	Primary school	3 (10%)	3 (10%)	0.501
	Secondary school	9 (30%)	12 (40%)	
	Diploma	10 (33.3%)	7 (23.3%)	
	Bachelor’s degree	8 (26.7%)	5 (16.7%)	
Occupation	Unemployed	13 (43.3%)	16 (53.3%)	0.0178
	Student	4 (13.3%)	2 (6.7%)	
	Self-employed	11 (36.7%)	9 (30%)	
	Government employed/Retired	2 (6.7%)	3 (10%)	
Residency	Metropolis	19 (63.3%)	13 (43.3%)	0.379
	Town/District	11 (36.7%)	17 (56.7%)	
Baseline YMRS	Mean ± SD	28.96 ± 5.30	32.06 ± 6.97	0.057

Normal Lithium Range (0.6–1.2 mEq/L)

#### Efficacy outcomes: primary outcome – change in YMRS score

As shown in Table 2, both the loading-dose lithium group (lithium loading dose plus quetiapine) and the standard-dose lithium group (standard lithium dose plus quetiapine) exhibited significant reductions in YMRS scores over the 14-day study period (*p* < 0.001 for within-group changes at all intervals). The loading-dose lithium group achieveda greater mean decrease from baseline at each time point (Day 3: Δ = −8.53 vs. −4.90; Day 7: Δ = −12.80 vs. −10.00; Day 14: Δ = −17.70 vs. −13.96; *p* < 0.001 for all comparisons). Direct comparisons of absolute scores between the groups at Days 3, 7, and 14, however, did not reveal statistically significant differences (*p* > 0.05 for all).

**Table 2:** Changes in YMRS scores in the loading-dose and standard-dose lithium groups over 14 days

YMRS score on Day	loading-dose lithium (Mean ± SD)	standard-dose lithium(Mean ± SD)	*p*-value
Day 0	32.06 ± 6.97	28.96 ± 5.30	0.057
Day 3	28.96 ± 5.30	23.53 ± 6.08	0.706
Day 7	24.06 ± 4.71	19.26 ± 5.38	0.818
Day 14	18.96 ± 4.63	14.36 ± 4.25	0.595

**Fig. 1:**
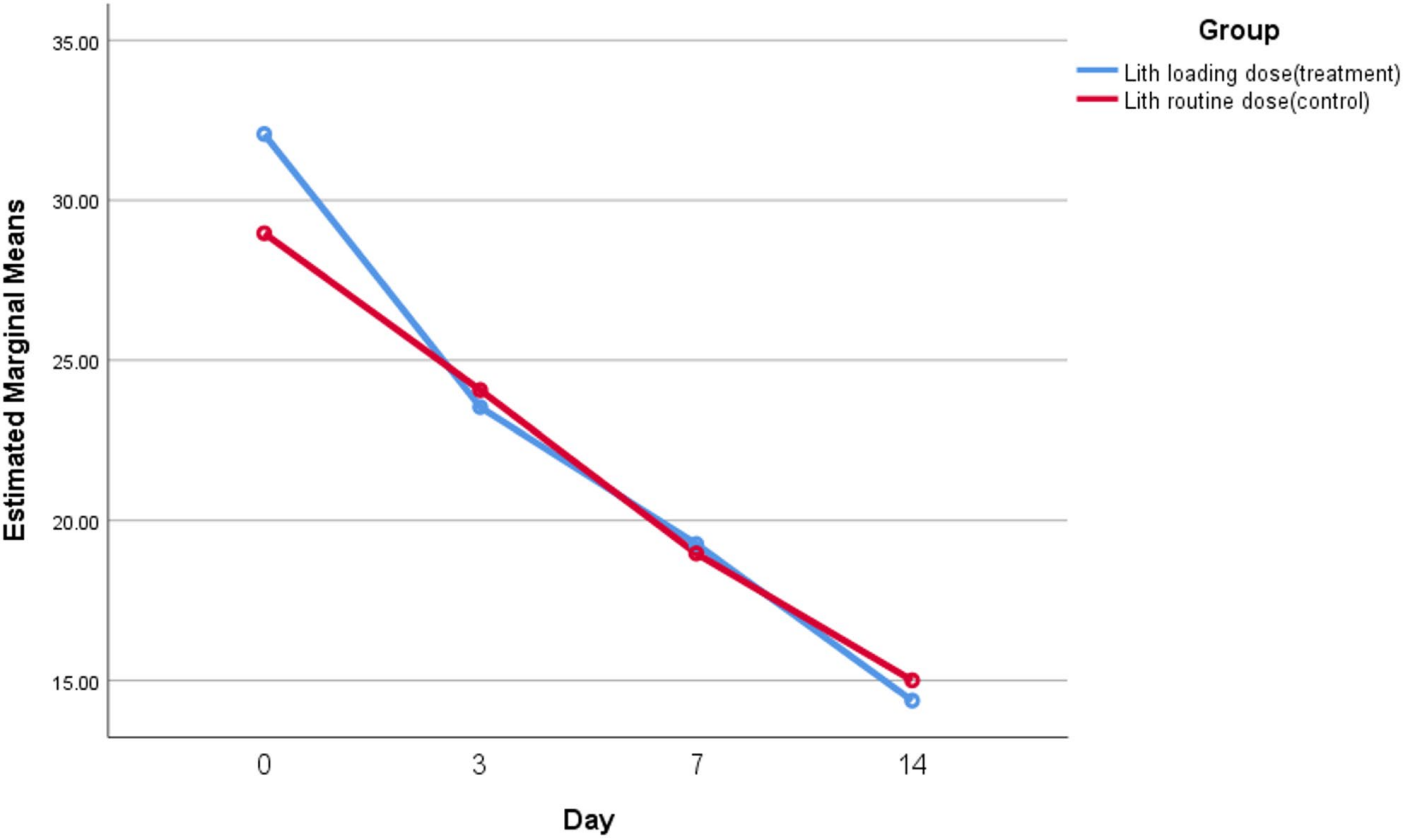
Estimated marginal mean YMRS scores for the loading-dose and standard-dose lithium groups across four assessment days (0, 3, 7, and 14)

Figure 1 illustrates the estimated marginal means of YMRS scores for both groups across Days 0, 3, 7, and 14, analyzed using the General Linear Model for repeated measures. Both groups showed progressive improvement, with the loading-dose lithium group demonstrating a steeper decline by Day 3, suggesting faster symptom resolution. By Day 7 and Day 14, scores converged between groups, consistent with the absence of significant differences in absolute values but supporting the greater cumulative reduction seen in the loading-dose lithium group. These graphical trends reinforce the tabulated data, highlighting the potential role of lithium loading in accelerating early therapeutic response during acute mania.

#### Safety and tolerability

In both groups, participants were closely monitored for potential adverse drug reactions, including electrocardiographic alterations, tremors, renal complications, changes in urinary output, hair loss, and dermatological symptoms. No such events were detected in either the loading dose or standard dose group at any assessment (Days 0, 3, 7, and 14). As shown in Table 3, the absence of observed adverse reactions precluded statistical comparisons of the incidence of drug-related side effects.

Aggressive and disruptive behavior scores did not differ significantly between groups on Days 0, 3, and 7. However, by Day 14, the loading dose group exhibited a significantly lower mean score compared with the standard dose group (*p* = 0.012), suggesting an enhanced effect of the loading regimen on behavioral control.

Longitudinal analysis revealed that reductions in aggressive/disruptive behavior scores from baseline were significantly greater in the loading dose group across all intervals assessed. As presented in Table 3, these differences reached statistical significance for changes from baseline to Day 3 (*p* = 0.001), Day 7 (*p* = 0.008), and Day 14 (*p* = 0.009), supporting the potential utility of the loading dose strategy in more rapidly attenuating aggressive tendencies in acute mania.

**Table 3:** Comparison of aggressive and disruptive behavior scores and their changes between loading-dose and standard-dose lithium groups

Day / Interval	loading-dose lithium (Mean ± SD)	standard-dose lithium(Mean ± SD)	*p*-value
Day 0 score	3.06 ± 1.79	2.40 ± 1.69	0.094
Day 3 score	1.40 ± 1.19	1.80 ± 1.21	0.210
Day 7 score	0.60 ± 0.93	1.06 ± 1.01	0.069
Day 14 score	0.06 ± 0.36	0.53 ± 0.89	0.012
Change to Day 3	−1.66 ± 1.18	−0.60 ± 1.30	0.001
Change to Day 7	−2.46 ± 1.71	−1.33 ± 1.76	0.008
Change to Day 14	−3.00 ± 1.72	−1.86 ± 1.90	0.009

## Discussion

This randomized clinical trial evaluated the efficacy and safety of a lithium carbonate loading dose regimen in combination with standard-dose quetiapine compared with a standard lithium titration approach plus quetiapine in patients presenting with acute mania. Over the 14-day study period, both treatment strategies resulted in significant symptomatic improvement, as reflected in YMRS scores.

It should be noted that the present trial was limited to a 14-day observation period, capturing only the acute phase of treatment response. While both regimens demonstrated significant symptom improvement within this timeframe, the findings do not inform on the sustainability of these effects or the comparative efficacy beyond the two-week mark. Long-term outcomes, including relapse prevention, functional recovery, and tolerability over time, remain to be established through extended follow-up studies.

However, the addition of a lithium loading dose did not provide a statistically significant benefit in either the speed or magnitude of symptom reduction compared with the standard titration regimen [11].

The observation that a loading dose did not significantly improve YMRS outcomes at Day 14 is particularly noteworthy. Although the rationale for a loading approach is to achieve therapeutic lithium levels more rapidly, potentially leading to faster symptom control, our results suggest that, when administered alongside quetiapine, the anticipated clinical advantage may not be realized within this timeframe. The rapid and robust antimanic effects of quetiapine, which have been demonstrated to produce significant improvements in YMRS scores within the first few days of treatment, could have substantially driven symptom reduction in both groups, thereby making any incremental benefit from faster lithium attainment [11–13].

The absence of substantial early superiority for the lithium-loading dose strategy in this acute inpatient setting is likely attributable to quetiapine’s rapid antimanic onset. This finding is corroborated by evidence from functional neuroimaging studies, which have shown that quetiapine can lead to a quicker normalization of limbic activation compared to lithium [6]. In situations where quetiapine is administered concurrently for acute mania, the benefit of accelerating lithium serum levels might be limited in terms of immediate symptom control.

Quetiapine dosing was consistent across study arms, titrated to 400–800 mg/day, ensuring that its potent antimanic effects were equally present across the sample. This uniformity may have minimized the influence of lithium dosing speed on short-term results. Moreover, adverse event rates—including nausea, tremor, and increased thirst—were comparable between groups. No patients were withdrawn due to serum lithium concentrations exceeding 1.4 mEq/L, indicating that careful monitoring and timely dosage adjustments allowed safe management in both regimens [7, 14, 15].

Our findings align with several prior studies that failed to demonstrate superior short-term efficacy for loading dose strategies, particularly in the context of combination therapy with other fast-acting antimanic agents. Lithium’s mood-stabilizing effects are generally considered to emerge gradually, and while rapid attainment of therapeutic serum levels is theoretically appealing, its clinical relevance in acute mania—especially alongside agents like quetiapine—requires cautious interpretation [4, 5, 11, 16].

Our results are consistent with pragmatic effectiveness trials that have reported comparable overall outcomes between lithium-based and quetiapine-based regimens over longer treatment durations (months), despite observed differences in time to peak therapeutic effect [9]. Furthermore, post-hoc analyses of maintenance-phase data consistently emphasize the critical role of maintaining lithium serum levels ≥ 0.6 mEq/L for effective relapse prevention [10]. This suggests that the sustained achievement of adequate lithium therapeutic targets, rather than the speed of initial attainment, may be a more influential factor in the long-term management of bipolar disorder.

One potential confounding factor is the occupational imbalance among participants. Acute mania often causes profound disruptions in daily functioning and professional responsibilities. While this investigation focused on symptom reduction, measures of functional recovery and reintegration into occupational roles might prove more sensitive in discerning the benefits of varying pharmacologic strategies [17–19].

The limited follow-up window represents another constraint. Although the study period adequately captures the acute phase of mania, longer-term comparative data—particularly with respect to relapse prevention and sustained mood stabilization—are lacking. It is plausible that differences in recovery rates might emerge over extended follow-up. Nonetheless, in clinical scenarios requiring short-term hospitalization and rapid symptom control, our findings indicate that standard lithium titration combined with quetiapine can be confidently employed to achieve effective outcomes without the need for a loading dose protocol [3, 4, 7].

Additional limitations include the absence of detailed data regarding illness history variables known to influence treatment response, such as age of onset, lifetime history of psychosis, number of prior mood episodes, and comorbid substance use. These variables were neither systematically collected nor controlled for in this analysis. Furthermore, although adverse events were comprehensively recorded for reported cases, the absence of a predefined and detailed checklist before trial initiation may have led to underreporting of some side effects [3, 8].

In summary, this study demonstrates that lithium loading in combination with standard-dose quetiapine does not confer significant short-term advantages over standard lithium titration with quetiapine in the management of acute mania. Both approaches are effective and well-tolerated, supporting the use of standard titration as an efficient and safe means of achieving rapid clinical improvement. Further research with extended follow-up and greater emphasis on functional recovery is warranted to determine whether long-term differences might emerge. Future multicenter studies should consider incorporating structured adverse event checklists and detailed illness history variables to facilitate more refined subgroup analyses. Given lithium’s gradual neuroprotective and mood-stabilizing effects [5] and quetiapine’s well-established rapid symptom relief [3] The standard titration approach for lithium remains a clinically sound and practical choice when both agents are employed for the acute management of mania.

## Conclusion

In this randomized trial, a lithium carbonate loading dose combined with standard-dose quetiapine did not provide short-term advantages over standard lithium titration with quetiapine in the treatment of acute mania. Both regimens achieved significant reductions in YMRS scores over 14 days, with similar safety profiles. The lack of added benefit from loading is likely attributable to quetiapine’s rapid antimanic effects and the gradual onset of lithium’s mood-stabilizing action. Given these findings, standard titration with quetiapine appears to be an effective and safe strategy for achieving rapid symptom control in the acute phase. Longer-term studies assessing relapse prevention, functional recovery, and the influence of illness history variables are warranted.

### Limitations

This single-center study with a short 14-day follow-up and modest sample size (*n* = 60) limits generalizability and hampers detection of rare or delayed lithium-related adverse effects. The absence of a placebo arm and partial blinding of medication administrators introduce potential bias, while significant baseline occupational imbalance (*p* = 0.0178) may confound results. A marginally higher baseline YMRS score in the loading-dose lithium group (*p* = 0.057) was acknowledged as a possible confounder, though statistical adjustments minimized its impact. These constraints highlight the need for larger, multicenter trials with longer follow-up to assess long-term and uncommon toxicities of high-dose lithium.

### Recommendations

In light of the findings of this trial, standard lithium titration combined with quetiapine should continue to be regarded as an effective and safe approach for managing acute mania in hospitalized patients, with no compelling evidence to support the routine use of a lithium loading protocol. Dose selection should be tailored to individual patient characteristics, including comorbidities and prior treatment responsiveness. Future research employing extended follow-up periods and incorporating comprehensive assessments of functional and social recovery, particularly the return to occupational roles, may provide a more nuanced understanding of potential differences between dosing strategies. Additionally, the systematic collection of detailed illness history variables and the use of standardized checklists for adverse event documentation will enhance the quality and generalizability of subsequent investigations.

## Data Availability

On reasonable request, the corresponding author is willing to provide the datasets used and analyzed during the present study.

## Abbreviations

YMRS: Young Mania Rating Scale
BD: Bipolar Disorder
BD-I: Bipolar Disorder Type I
BD-II: Bipolar Disorder Type II
C: Control
LCLPQ: Lithium Carbonate Loading Dose plus Quetiapine
LPLC: Lithium Loading Dose plus Quetiapine
PL: Placebo
RCT: Randomized Controlled Trial
ECG: Electrocardiogram
H1: Histamine receptor type 1
α1, α2: Alpha-adrenergic receptors (alpha-1 and alpha-2)
5-HT1A, 5-HT2A: Subtypes of serotonin receptors (5-hydroxytryptamine receptor type 1 A and type 2 A)
DSM-V: Diagnostic and Statistical Manual of Mental Disorders, Fifth Edition
SPSS: Statistical Package for the Social Sciences
mEq/L: Milliequivalents per liter
P: Probability Value (used in statistical analysis)
mg: Milligram
kg: Kilogram
hr: Hour (related to half-life measurements)

## References

[1] Kawa ADDINENREFLIST, Carter I, Joyce JD, Doughty PR, Frampton CJ, Elisabeth Wells CM. Gender differences in bipolar disorder: age of onset, course, comorbidity, and symptom presentation. Bipolar Disord. 2005;7(2):119–25. 15762852 10.1111/j.1399-5618.2004.00180.x

[2] Grande I, Berk M, Birmaher B, Vieta E. Bipolar disorder. Lancet. 2016;387(10027):1561–72. 26388529 10.1016/S0140-6736(15)00241-X

[3] Yatham LN, Kennedy SH, Parikh SV, Schaffer A, Bond DJ, Frey BN, et al. Canadian Network for Mood and Anxiety Treatments (CANMAT) and International Society for Bipolar Disorders (ISBD) 2018 guidelines for the management of patients with bipolar disorder. Bipolar Disord. 2018;20(2):97–170. 29536616 10.1111/bdi.12609 PMC5947163

[4] Malhi GS, Gessler D, Outhred T. The use of lithium for the treatment of bipolar disorder: recommendations from clinical practice guidelines. J Affect Disord. 2017;217:266–80. 28437764 10.1016/j.jad.2017.03.052

[5] McKnight RF, Adida M, Budge K, Stockton S, Goodwin GM, Geddes JR. Lithium toxicity profile: a systematic review and meta-analysis. Lancet. 2012;379(9817):721–8. 22265699 10.1016/S0140-6736(11)61516-X

[6] Patino LR, Klein CC, Strawn JR, Blom TJ, Tallman MJ, Adler CM, et al. A Randomized, Double-Blind, Controlled Trial of Lithium Versus Quetiapine for the Treatment of Acute Mania in Youth with Early Course Bipolar Disorder. J Child Adolesc Psychopharmacol. 2021;31(7):485–93. 34520250 10.1089/cap.2021.0039 PMC8568789

[7] Vieta E, Suppes T, Eggens I, Persson I, Paulsson B, Brecher M, et al. Efficacy and safety of quetiapine in combination with lithium or divalproex for maintenance of patients with bipolar I disorder (international trial 126). J Affect Disord. 2008;109(3):251–63. 18579216 10.1016/j.jad.2008.06.001

[8] Hui T, Kandola A, Shen L, Lewis G, Osborn D, Geddes J, et al. A systematic review and meta-analysis of clinical predictors of lithium response in bipolar disorder. Acta psychiatrica Scandinavica. 2019;140(2):94–115. 31218667 10.1111/acps.13062 PMC6772083

[9] Nierenberg AA, McElroy SL, Friedman ES, Ketter TA, Shelton RC, Deckersbach T, et al. Bipolar CHOICE (Clinical Health Outcomes Initiative in Comparative Effectiveness): a pragmatic 6-month trial of lithium versus quetiapine for bipolar disorder. J Clin Psychiatry. 2016;77(1):90–9. 26845264 10.4088/JCP.14m09349

[10] Nolen WA, Weisler RH. The association of the effect of lithium in the maintenance treatment of bipolar disorder with lithium plasma levels: a post hoc analysis of a double-blind study comparing switching to lithium or placebo in patients who responded to quetiapine (Trial 144). Bipolar Disord. 2013;15(1):100–9. 23228201 10.1111/bdi.12027

[11] McKnight RF, Chesney E, Amit BH, Geddes J, Cipriani A. Lithium for acute mania. Cochrane database Syst reviews. 2019(6). 10.1002/14651858.CD004048.pub4 PMC6544558 31152444

[12] Vieta E, Mullen J, Brecher M, Paulsson B, Jones M. Quetiapine monotherapy for mania associated with bipolar disorder: combined analysis of two international, double-blind, randomised, placebo-controlled studies. Curr Med Res Opin. 2005;21(6):923–34. 15969892 10.1185/030079905X46340

[13] Ketter TA, Miller S, Dell’Osso B, Wang PW. Treatment of bipolar disorder: Review of evidence regarding quetiapine and lithium. J Affect Disord. 2016;191:256–73. 26688495 10.1016/j.jad.2015.11.002

[14] Bauer M, Gitlin M. The essential guide to lithium treatment. Springer; 2016.

[15] Kim TT, Dufour S, Xu C, Cohen ZD, Sylvia L, Deckersbach T, et al. Predictive modeling for response to lithium and quetiapine in bipolar disorder. Bipolar Disord. 2019;21(5):428–36. 30729637 10.1111/bdi.12752

[16] Geddes JR, Miklowitz DJ. Treatment of bipolar disorder. lancet. 2013;381(9878):1672–82. 23663953 10.1016/S0140-6736(13)60857-0 PMC3876031

[17] Tohen M, Frank E, Bowden CL, Colom F, Ghaemi SN, Yatham LN, et al. The International Society for Bipolar Disorders (ISBD) Task Force report on the nomenclature of course and outcome in bipolar disorders. Bipolar Disord. 2009;11(5):453–73. 19624385 10.1111/j.1399-5618.2009.00726.x

[18] Goldstein BI, Birmaher B, Carlson GA, DelBello MP, Findling RL, Fristad M, et al. The International Society for Bipolar Disorders Task Force report on pediatric bipolar disorder: Knowledge to date and directions for future research. Bipolar Disord. 2017;19(7):524–43. 28944987 10.1111/bdi.12556 PMC5716873

[19] Van der Voort TY, Seldenrijk A, van Meijel B, Goossens PJ, Beekman AT, Penninx BW, et al. Functional versus syndromal recovery in patients with major depressive disorder and bipolar disorder. J Clin Psychiatry. 2015;76(6):7517. 10.4088/JCP.14m09548 26132690

